# Acute myocardial infarction from a lower-middle income country—A comprehensive report on performance measures and quality metrics using National Cardiovascular Data Registry

**DOI:** 10.1371/journal.pone.0294396

**Published:** 2023-11-15

**Authors:** Farhala Baloch, Ainan Arshad, Sher Muhammad Sethi, Javed M. Tai

**Affiliations:** 1 Adult Cardiology, Department of Medicine, Aga Khan University Hospital, Karachi, Sindh, Pakistan; 2 Internal Medicine, Department of Medicine, Aga Khan University Hospital, Karachi, Sindh, Pakistan; University of Bologna, ITALY

## Abstract

**Introduction:**

Epidemic of cardiovascular disease (CVD) is widely projected in South Asian population and estimated to get double in two decades. Ischemic heart disease (IHD) is one of the spectrums of CVD and acute myocardial infarction (AMI) being the common manifestations of IHD. National Cardiovascular Data Registry (NCDR) is a registry data that measure their practices and improve quality of care. In this project we aim to see our performance trends in the care of IHD including AMI patients over two year’s period.

**Material & methods:**

A cross sectional study conducted at the Aga Khan University Hospital, Karachi, Pakistan. All patients aged 18 years and above admitted to adult Cardiology units with chest pain and acute coronary syndrome are eligible to be included in NCDR data set. Data on demographics and initial characteristics of patients were extracted from NCDR institutional dataset. The data was then compared between 2019 and 2020 on performance, quality, and efficiency metrics.

**Result:**

In 2019 to 2020, 1542 patients with acute coronary syndrome and stable ischemic heart disease were admitted. Out of these, 1042 patients (67.8%) were males. According to our data, the 2020 mortality rate was about 5.25%. In 2019 and 2020, bleeding rates were 1.1% and 1.6%, respectively. Our data showed 100% PCI in 90 minutes in 2019 while 87% in 2020. According to the appropriateness criteria for PCI, 80% were appropriate, while 20% were possibly appropriate in both years. The median length of stay following a procedure was 2 days in 2019 and 1 day in 2020.

**Conclusion:**

This study described the common and unique characteristics of patients with myocardial infarction representing population from South Asian region. Overall, the procedural performance measure and outcome metrics are up to the international benchmarks. Cultural, financial, and pandemic effects identified certain challenges.

## Introduction

Epidemic of cardiovascular disease (CVD) is widely projected in South Asian population and estimated to get double in two decades [1]. Ischemic heart disease (IHD) is one of the spectrums of CVD and acute myocardial infarction (AMI) being the common manifestations of IHD. ST elevation myocardial infarction (STEMI) and Non-ST elevation myocardial infarction (NSTEMI) are the two clinical presentations in AMI [2, 3]. STEMI is a common presentation accounting for up to 25%–40% of all MI cases brought to the hospital, with up to 7%– 10% in-hospital mortality documented in developed countries and up to 9.6% in developing country [4, 5]. It results from complete or near complete occlusion of one of the coronary arteries and can results in sudden disruption of the blood supply to myocardium. American College of Cardiology and American Heart Association (ACC/AHA) recommend timely revascularization of the culprit coronary artery to save myocardium [6]. NSTEMI although does not results due to a complete occlusion but the fourteen days and thirty days mortality of NSTEMI may equals to or more than in STEMI patients.

The management of AMI patients begins in emergency department or first medical contact (FMC) and is continued in specialized cardiac care units till the patients is discharge home. It is a process of care followed globally. Various quality parameters are identified, taken care of and benchmark during the process of care for AMI patients to deliver best and defect free care. ECG within ten minutes of arrival, aspirin within 60 minutes of arrival and achieving a door to balloon time of less than 90 minutes are the set quality process indicators. Similarly, overall and risk adjusted mortality of AMI patients, risk adjust bleeding, acute kidney injury, stroke is some of the major adverse events and outcome indicators measures as key quality metrics globally [7, 8].

National Cardiovascular Data Registry (NCDR) is the suit of eight registries established by ACC/AHA and endorsed by Society for Cardiovascular Angiography and Intervention (SCAI) [9] as a complex network of cardiovascular experts taking care of AMI patients to help them measure their practices and improve quality of care. In this project we aim to see our performance trends in the care of AMI patients over two years period, immediately and one year later to joining registry. We aim to identify challenges specific to our context being an institute in a lower middle-income country.

## Material and methods

### Study design

A cross sectional study conducted at the Aga Khan University Hospital (AKUH), Karachi, Pakistan. AKUH is one of the few Joint Commission International Accreditation (JCIA) accredited tertiary care hospitals. Ethical exemption (Ethical Review Committee Number: 2021-6665-18999) was obtained from the institution’s Ethical Review Committee and consent was waived. Our institution participates with NCDR as a quality improvement project for the care of AMI patients.

### Patient population

All patients aged 18 years and above admitted to adult Cardiology units with chest pain and acute coronary syndrome are eligible to be included in NCDR data set for two registries Cath-PCI and Chest pain-MI. The study included all the adult patients who were admitted with chest pain in the year 2019–2020.

### NCDR registry

As a retrospective study, we use the NCDR data from January 2019 to December 2020. Two out of eight hospital-based registries named CATH-PCI registry and CHEST PAIN-MI registry includes all adults’ patients ages 18 years and above with cardiovascular disease including AMI, unstable angina, low risk chest pain and elective cardiovascular procedures including diagnostic coronary angiogram and percutaneous coronary intervention. Each registry featuring standardized, evidence-based data elements and definitions. NCDR data holds answers to complex questions about patient risk factors and outcomes, procedure and treatment trends and adherence to guidelines. The results of key performance measures and quality metrics is shared by registry as online reports with the registry site manager (RSM) and registry director of the participating hospitals. The raw data can be extracted for the self-analysis and result generation by the registry representative of the institute using dedicated login identification details. This data can be used to answer further research questions.

### Data collection

Our institute only provide baseline data to NCDR data base. Medicine and cardiology trainees routinely fill these forms for all the patients during the index hospitalization and completion of forms is checked by a research assistant upon patient discharge from the hospital. Research assistant enters all this information into the electronic database on timely basis. This data is regularly uploaded to the NCDR using the paid online NCDR data entry dashboard. This whole process is supervised by a registry site manager.

Data on demographics and initial characteristics of patients were extracted as it is recorded in NCDR dataset. These include their past risk factors, smoking status, prior New York Heart Association (NYHA) classification and heart failure types. Angina was broadly categorized into typical, atypical, and non-angina chest pains. Typical anginal pain was defined as squeezing and pressure sensation at mid chest region with radiation, exacerbated on exertion and associated with shortness of breath while atypical angina was defined as chest pain with light headedness, nausea and dizziness with associated fatigue and weakness [10]. Non-angina chest pains incudes those who have chest pain with symptoms of gastro-esophageal reflux disease, psychiatric illness and or any musculoskeletal disease [11]. Electrocardiograms details were noted for all of these patients.

Prior to the left heart catheterization, various laboratory parameters were recorded that include troponin, hemoglobin, pre and post procedure serum creatinine levels. During the procedure, systolic blood pressure, contrast volume and fluoroscopy time were also documented. Angiographic preference (diagnostic versus therapeutic) was also identified. Access site used for coronary angiography, indication for angiography, dominant vessels and vessel stenosis were observed and recorded. In hospital mortality and discharge disposition were noted.

### Outcomes

The data was then compared between 2019 and 2020 on performance, quality, and efficiency metrics. Performance measures consist of mortality, bleeding, and medications given at discharge. Quality measures include median time for PCI, PCI within 90 minutes, prescribing aspirin, P2Y12 inhibitor, and statins on discharge, rehabilitation referral and post procedure creatinine levels. Efficiency measures include appropriateness criteria for PCI and length of hospital stay.

### Statistical methods

IBM Statistical Package for the Social Sciences (SPSS) Version 26 (IBM Corp., Armonk, USA) was used for data analysis. Frequency and percentages were used to express categorical variables. The mean and standard deviation were used to express quantitative variables. Categorical variables were compared by a chi-square test with a significance level of <0.05. Quantitative variables were compared with an independent sample t-test with a significance level of <0.05. A standard benchmark was recorded and a comparison between 2019 and 2020 was done with frequencies and percentages.

## Results

In 2019 to 2020, 1542 patients were registered in the NCDR registry. Out of these, 1006 were registered in CATH-PCI while 536 patients were registered in CHEST PAIN-MI registry. From total of 1542 patients, 1042 patients (67.8%) were men. Hypertension and diabetes were the major risk factors which were present in 1041 (67.5%) and 712 (46.2%) patients respectively. 56 patients (3.6%) were active smokers and 44 patients (2.9%) were ex-smokers. Nine patients (0.6%) had a cardiac arrest outside the hospital before admission. 64 (4.2%) of the patients had New York Heart Association (NYHA) functional classification II. Patients with systolic dysfunction were 48 (3.1%), while those with diastolic dysfunction were 16 (1%) patients. The following Table 1 summarizes the characteristics of the patients before admission to the hospital.

**Table 1:** Prior characteristics of patients.

	N (%)
**Mean Age ± S.D.**	61.5 ± 12.6
**Sex**	
• Male	1042 (67.8)
• Female	494 (32.2)
**Risk Factors**	
• Hypertension	1041 (67.5)
• Diabetes	712 (46.2)
• Dyslipidemia	360 (23.3)
• Past history of MI	96 (6.2)
• Family history of CAD	31 (2.0)
• History of cardiovascular disease	24 (1.6)
• History of heart failure	104 (6.7)
• History of chronic lung disease	18 (1.2)
• End stage renal disease	5 (0.3)
• Prior PCI	95 (6.2)
• Prior PAD	6 (0.3)
• Prior CABG	48 (3.1)
**Smoking**	
• Never	1419 (92)
• Former	44 (2.9)
• Current–Everyday	45 (2.9)
• Current–Some days	4 (0.3)
• Current—Unknown	7 (0.5)
• Unknown if ever smoked	11 (0.7)
**Prior NYHA**	
• Unknown	1438 (93.3)
• Class I	4 (0.3)
• Class II	64 (4.2)
• Class III	31 (2.0)
• Class IV	5 (0.3)
**Heart Failure Type**	
• Unknown	1478 (95.8)
• Systolic	48 (3.1)
• Diastolic	16 (1.0)

**Fig 1:**
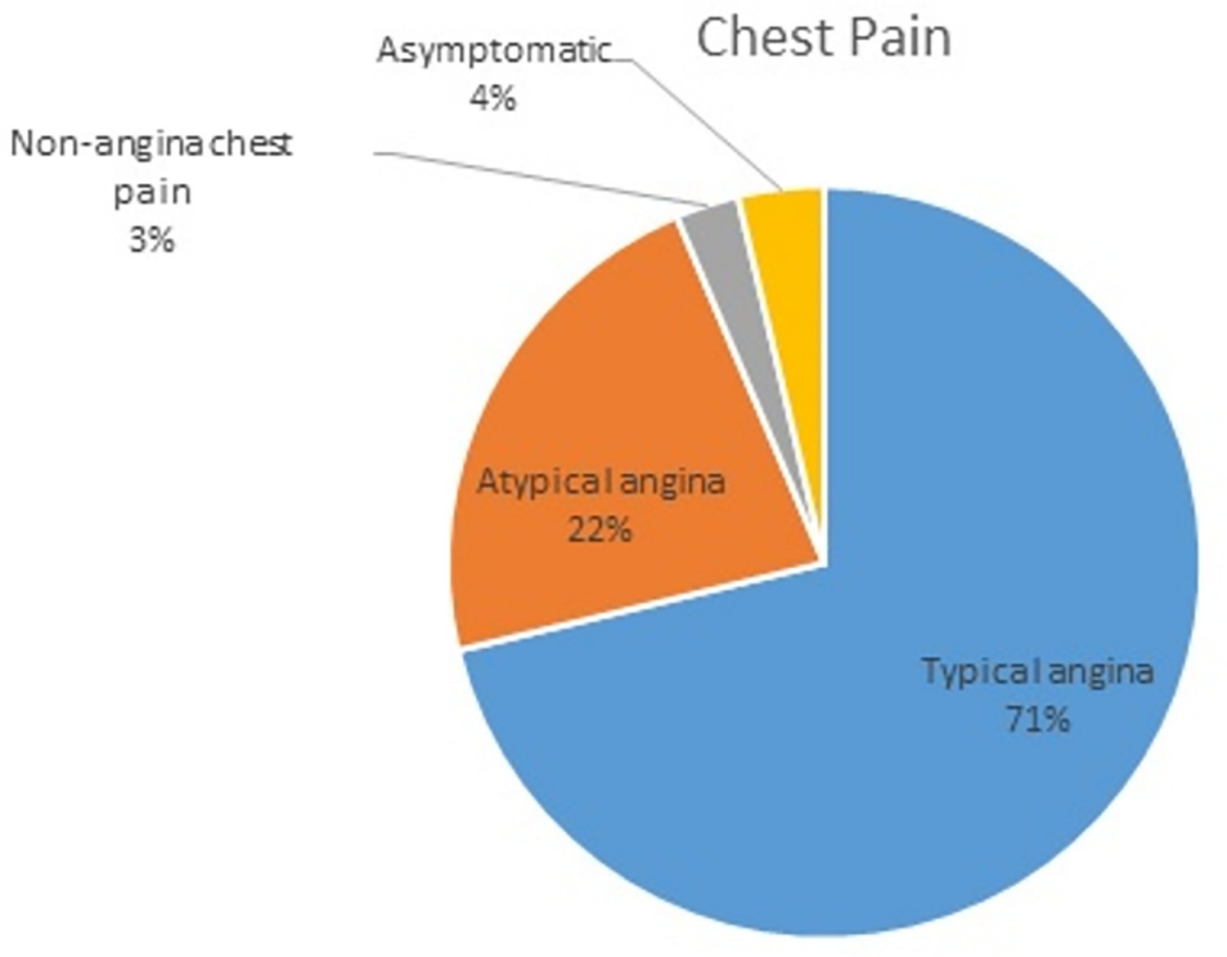
Types of complaints on presentation.

**Fig 2:**
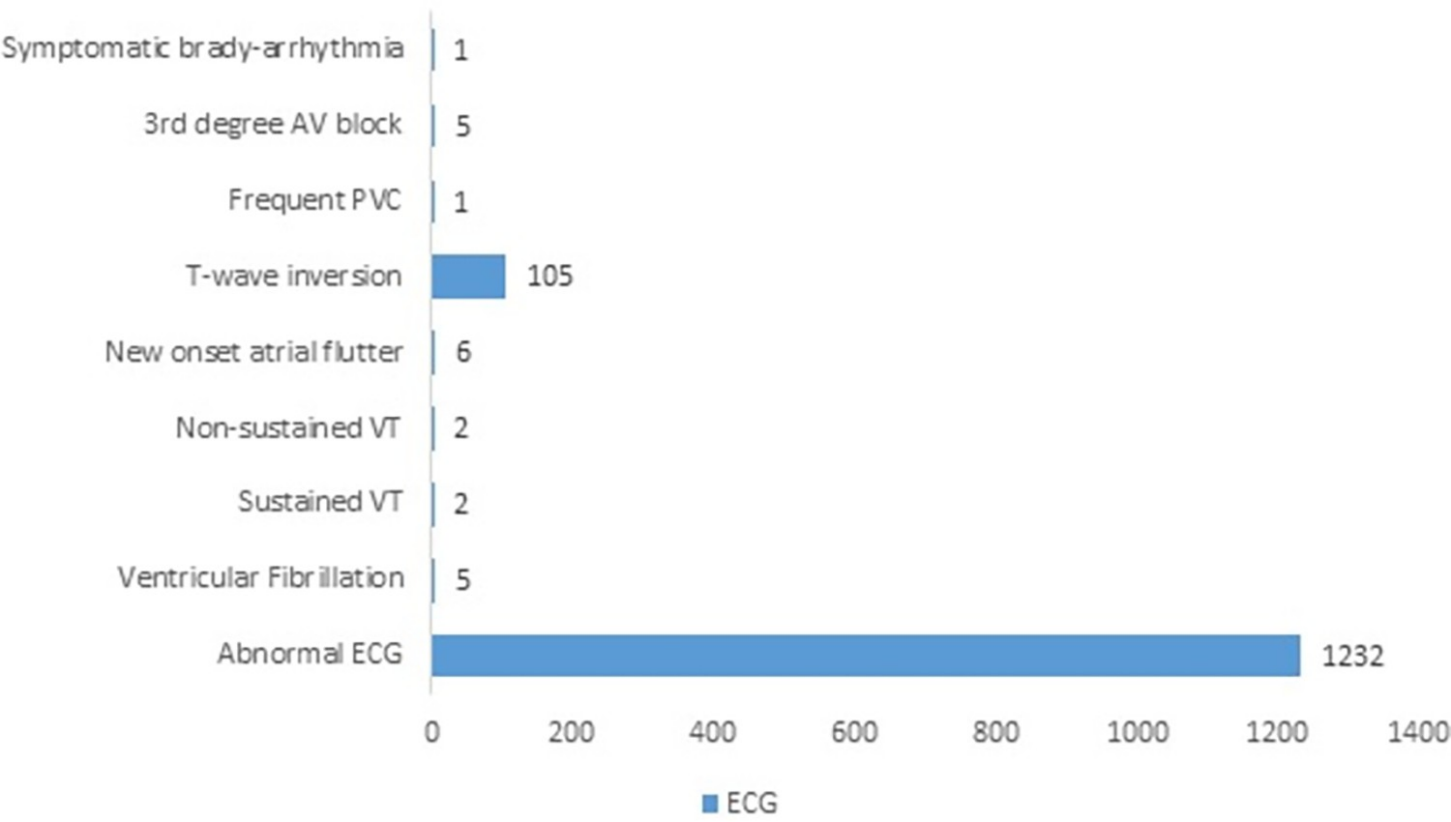
Electrocardiographic changes identified in the patients on admission.

The presenting complaints were typical angina chest pain in 1099 (71.3%) patients. An illustration of the different types of complaints presented by patients is shown in Fig 1. Five patients (0.3%) had cardiovascular instability on the initial encounter. 1232 patients (78.6%) had abnormal electrocardiograms. In Fig 2, electrocardiographic patterns from the patients are represented.

In CHEST PAIN-MI registry 536 patients were enrolled. Out of these, 415 (77.4%) patients had NSTEMI, 14 (2.6%) patients had unstable angina, 32 (6%) patients had low-risk chest pain while 75 (14%) patients had STEMI. A total proportion of PCI in these 536 patients were in 188 patients (35%). From 415 NSTEMI patients, 136 patients (32.7%) had an early PCI within the first day of the admission. In 75 STEMI patients, 37 patients (49.3%) had an immediate PCI. Stent was implanted in 167/188 patients (88.8%). All the patients received drug-eluting stent.

A total of 1534 (99.5%) patients underwent diagnostic coronary angiography. Among these, 429 patients (27.8%) underwent percutaneous intervention. The radial artery was used for coronary angiography in 1329 (86.2%) of the patients. Acute ischemic syndrome in less than or within 24 hours was the primary reason for diagnostic coronary angiography in 1048 (68%) patients and for more than 24 hours in 168 (10.9%) patients. Angiograms showed right-sided dominance in 1356 patients (87.9%). There were 1143 (74.1%) patients had normal vessel stenosis, while three (0.2%) patients had graft vessel stenosis. Most of the patients (95.8%) were discharged to their homes while 4.2% died in hospital. Table 2 depicts pre-procedural blood pressures, laboratory investigations and coronary angiography parameters.

**Table 2:** Left heart catheterization procedural parameters.

	*Mean ± S*.*D*.
**Mean Procedural Systolic Blood Pressure (mmHg)**	136 ± 13
**Mean Contrast Volume (ml)**	48 ± 26
**Mean Fluoroscopy time (minutes)**	5.9 ± 7.7
**Pre-procedure troponin (ng/ml)**	9.72 ± 23.85
**Pre-procedure creatinine (mg/dL)**	1.11 ± 0.66
**Pre-procedure hemoglobin (g/dL)**	12.9 ± 1.9
**Post-procedure creatinine (mg/dL)**	1.19 ± 0.84
	***N (%)***
**Diagnostic Coronary Angiography**	1534 (99.5)
**Percutaneous Intervention**	429 (27.8)
**Access Site**	
○ Radial	1329 (86.2)
○ Femoral	208 (13.5)
○ Brachial	4 (0.3)
○ Other	1 (0.1)
**Cath Lab visit indications**	
○ ACS ≤ 24 hours	1048 (68)
○ ACS > 24 hours	168 (10.9)
○ New onset angina ≤ 2 months	103 (6.7)
○ Worsening angina	119 (7.7)
○ Resuscitated cardiac arrest	1 (0.1)
○ Stable known CAD	4 (0.3)
○ Suspected CAD	46 (3.0)
○ Valvular disease	10 (0.6)
○ Cardiac arrhythmia	1 (0.1)
○ Cardiomyopathy	8 (0.5)
○ LV dysfunction	10 (0.6)
**Dominance**	
○ Right	1356 (87.9)
○ Left	122 (7.9)
○ Co-dominance	64 (4.2)
**Stenosis**	
○ Normal vessel	1143 (74.1)
○ Graft vessel	3 (0.2)
**Discharge (Alive)**	1407 (91.3)
**Mortality**	65 (4.2)
**Medicine reconciliation**	1475 (95.7)

**Fig 3:**
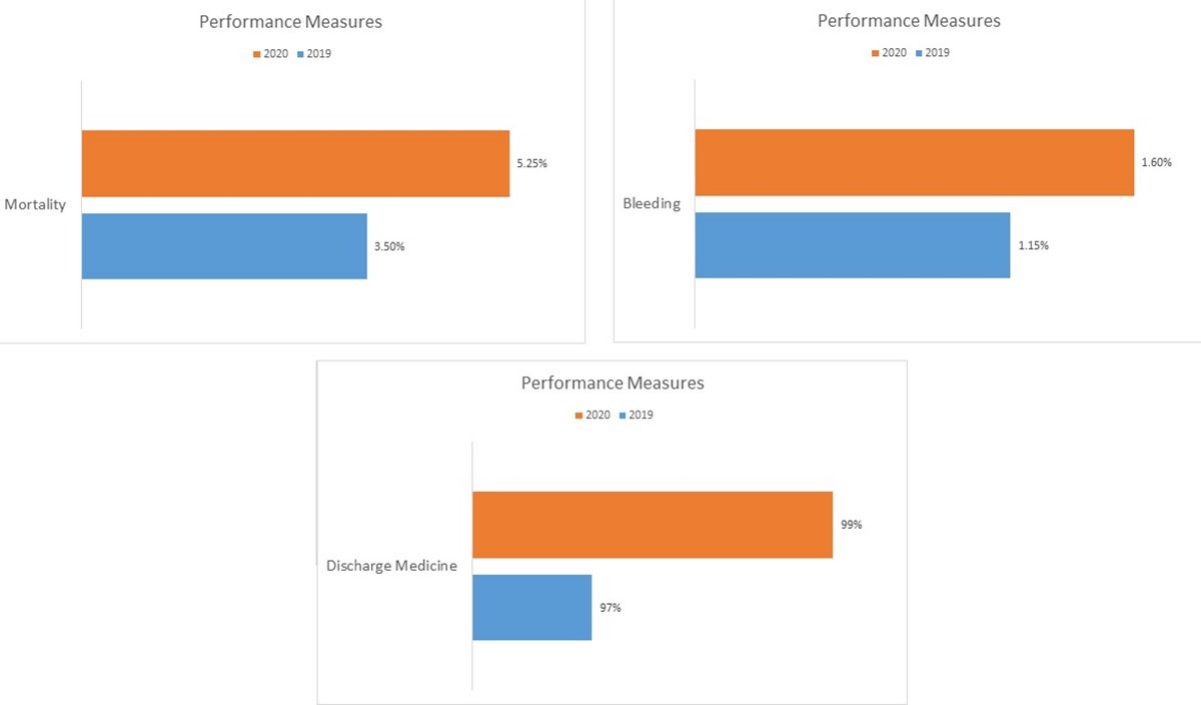
Comparison of performance measure between 2019 and 2020.

A standard benchmark for mortality is 3.5% per year. According to our data, the 2020 mortality rate was about 5.25%. Benchmark bleeding rate is 1.4%. In 2019 and 2020, bleeding rates were 1.1% and 1.6%, respectively. The discharge medicine benchmark is 98% and we had a rate of 99% in 2020. Fig 3 shows the comparison of performance measures in 2019 and 2020.

**Fig 4:**
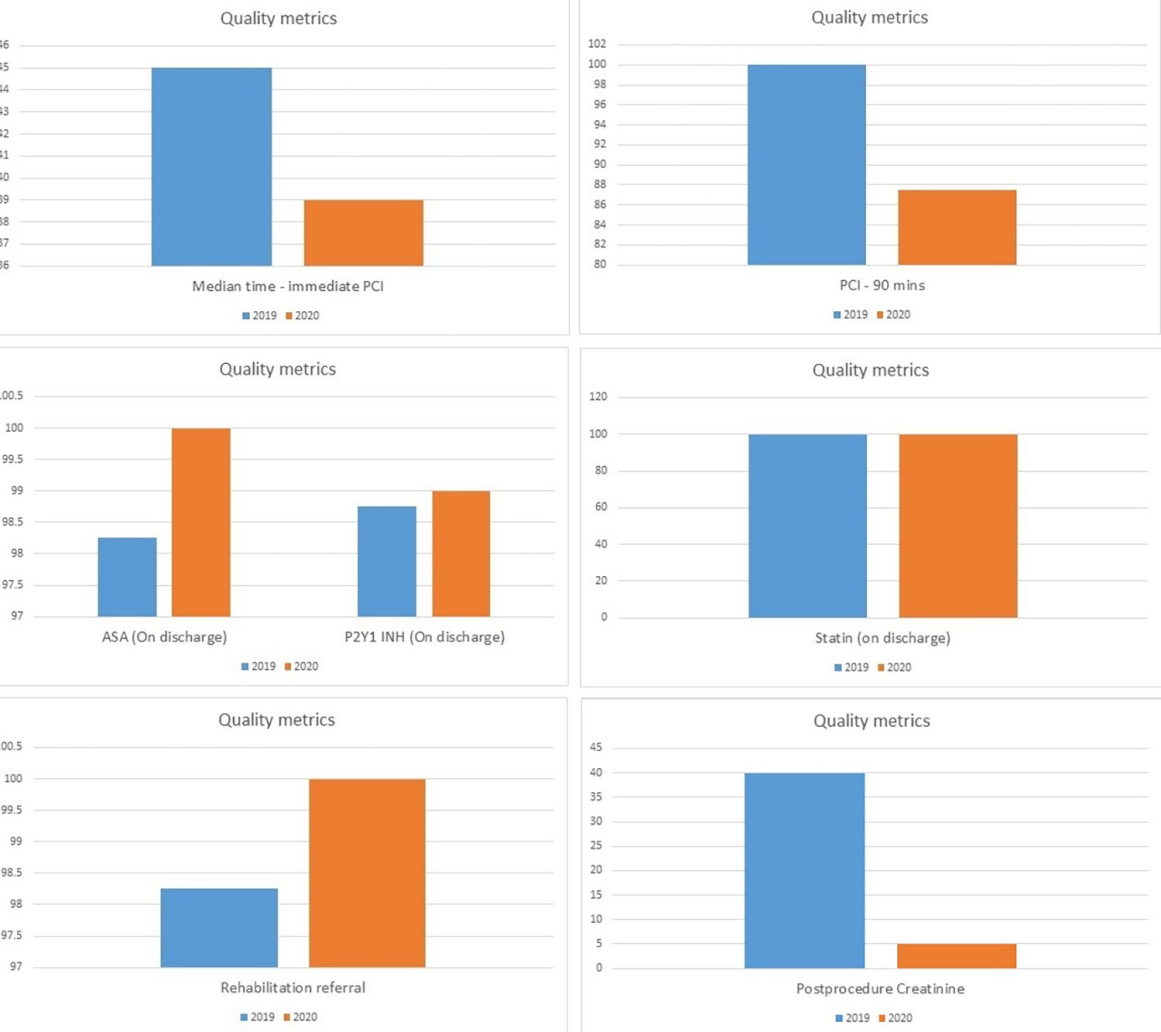
Quality metrics observed in 2019 and 2020.

Quality metrics include different factors which have been shown in Fig 4. The recommended benchmark for median time for immediate percutaneous intervention is 60 minutes. We had a median time for immediate PCI of 45 minutes in 2019 and 30 minutes in 2020. The standard benchmark for PCI in 90 minutes is 95%. Our data showed 100% PCI in 90 minutes in 2019 while 87% in 2020. The benchmark for aspirin and P2Y12 inhibitor on discharge is 99% while for statin is 100%. On discharge, aspirin was given to 98.25% in 2019 and 100% in 2020, while P2Y12 inhibitors were given to 99.25% in 2019 and 99% in 2020. 100% of patients received statins on discharge in both years. The benchmark for rehabilitation referral is 100%. When compared from 2019 to 2020, 98.25% referred in 2019 and 100% in 2020. Benchmark for checking post-procedure creatinine is 90%. A 45% creatinine measurement was taken in 2019 and only a 5% measurement in 2020.

**Fig 5:**
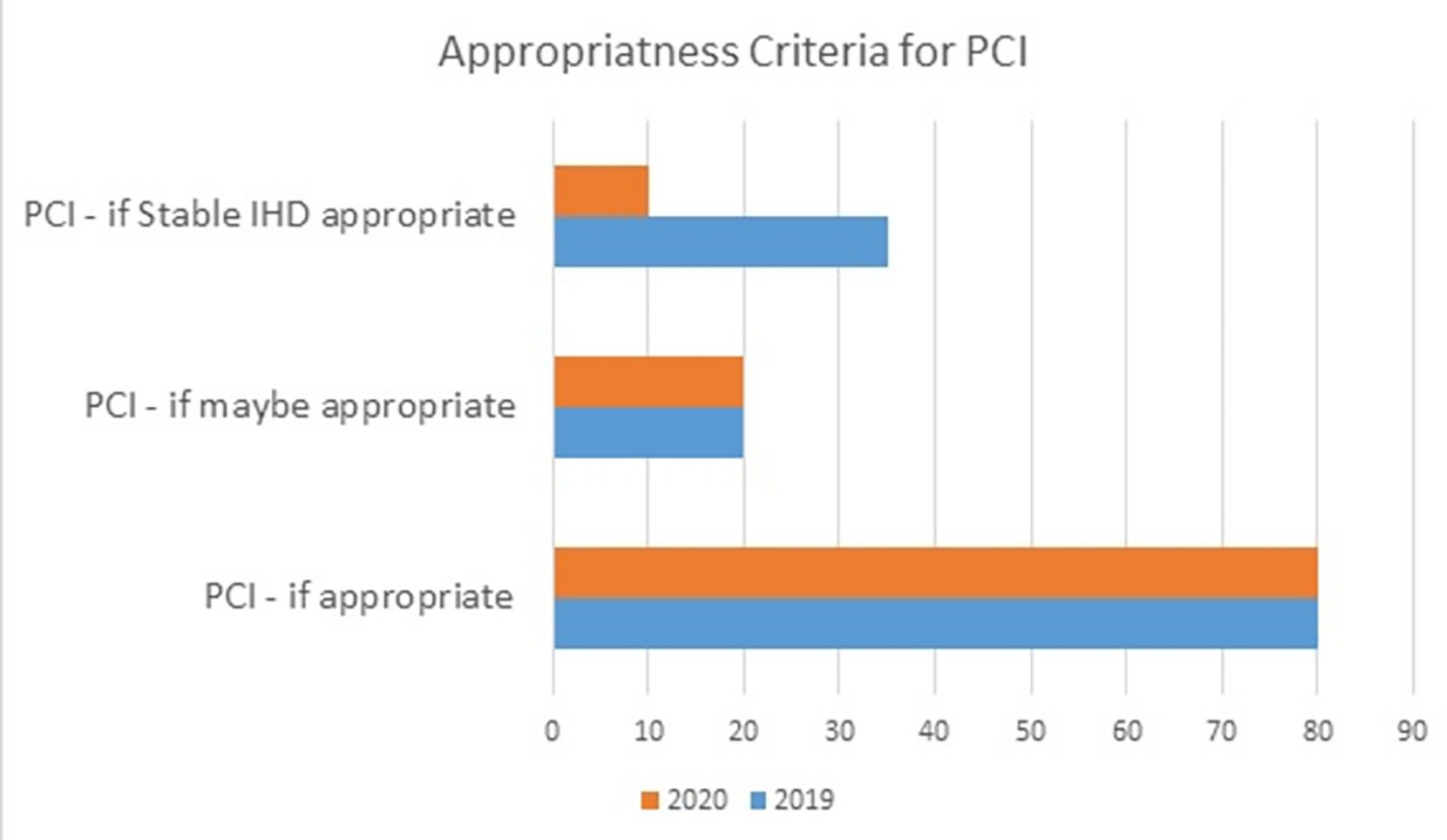
Appropriateness criteria for PCI between 2019 and 2020.

According to the appropriateness criteria for PCI, 80% were appropriate, while 20% were possibly appropriate in both years. A decline in PCI appropriateness for stable ischemic heart disease was identified in 2020 of around 10%. Fig 5 compares the appropriateness criteria for PCI in 2019 and 2020.

**Fig 6:**
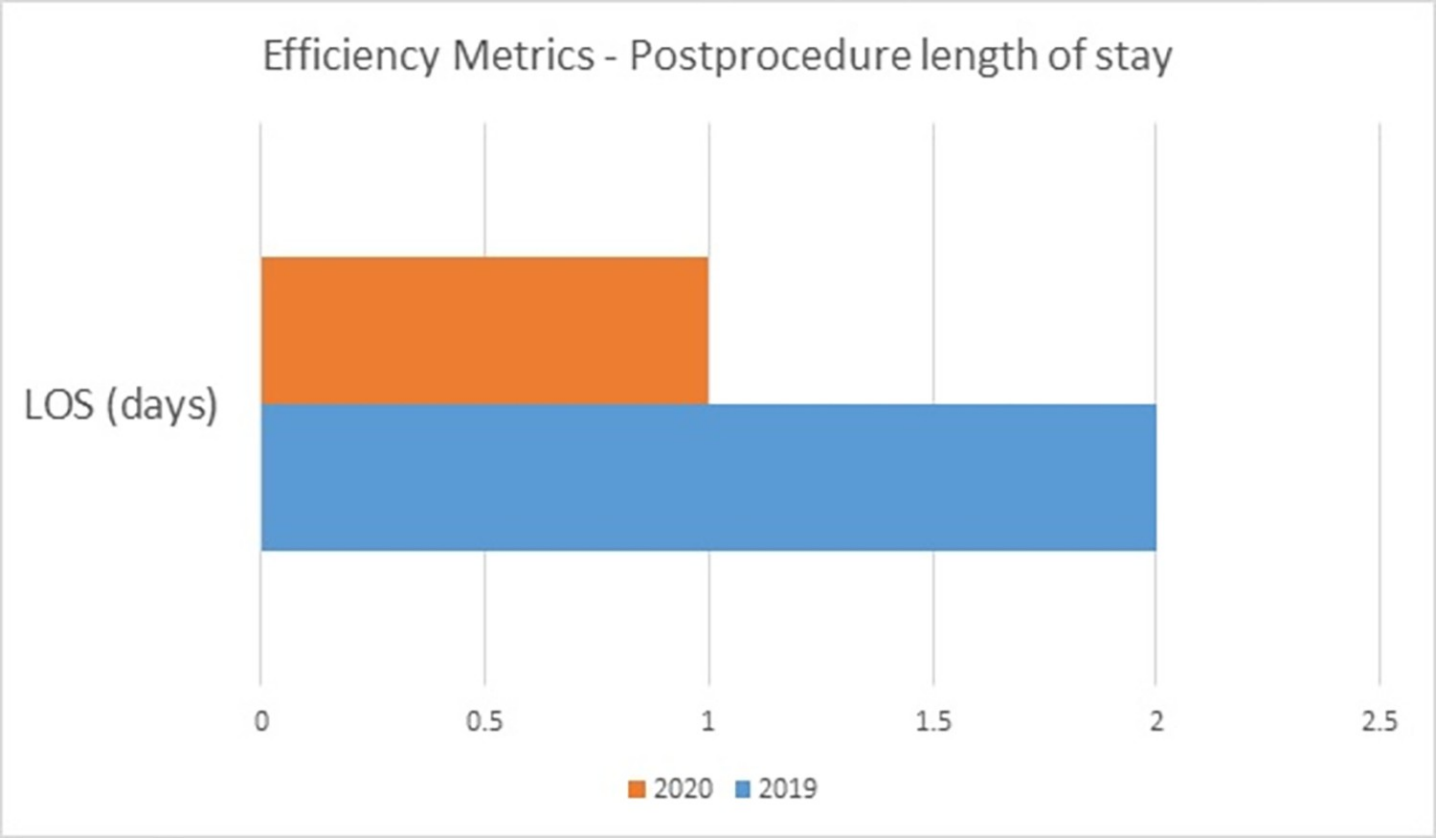
Post-procedure length of hospital stay in 2019 and 2020.

Post-procedure length of hospital stay is used as an efficiency metric by NCDR. As shown in Fig 6, the median length of stay following a procedure was 2 days in 2019 and 1 day in 2020.

Our analysis compared the characteristics of survivors and non-survivors. The mean age of survivors was 61 years and non-survivors was 65 years (p-value: 0.02). Non-survivors were more likely to have a history of cardiovascular disease (4.6%) compared to survivors (1.4%) (p-value: 0.04). Pre-procedure mean hemoglobin levels were 10.2 ± 1.9 (p-value: <0.001) and mean creatinine levels were 1.8 ± 0.6 (p-value: 0.002) in non-survivors. The PCI was performed in 420 patients (28.4%) who survived versus 43 patients (66.6%) who did not. (p-value: 0.009) (S1 Table).

## Discussion

In summary, we had 1542 patients who were admitted with coronary disease from 2019 to 2020 in cardiology unit and we registered them in the NCDR. Majority of them were male gender (67.8%). The major risk factor in our study population was diabetes and hypertension. Almost all 99.5% of patients underwent diagnostic coronary angiography and 27.8% had percutaneous intervention. 94.5% of the patients were successfully discharged alive from the hospital. Performance measures were well in the standard benchmark in 2019 but were slightly higher in 2020. Most of the quality metrics in 2020 were according to the standard recommendations. Efficiency was measured using post-procedure length of hospital stay and it was 2 days in 2019 and decrease to 1 day in 2020. The decline in performance and shortened in length of stay could be due to the COVID-19 pandemic in 2020.

The American College of Cardiology had designed an NCDR program to serve as a basis of quality improvement for the past 20 years. This program had been flourished in past few years as it sets standard benchmarks for quality measures [12]. NCDR are now being established as a standard project in all well recognized health care institutes and hospitals. This data had resulted in recognizing deficiencies and improving quality measures in the hospital settings [13]. Aga Khan University Hospital is one of the tertiary care hospital in Pakistan which is more focused in promoting quality care to the patients. To ensure this, our hospital is a registered in both the registry (Cath-PCI and Chest pain-MI registry).

Acute coronary syndrome (ACS) had a higher incidence in South Asian population as compare to other ethnic groups [14]. Rashid et al. had reported 57% incidence of ACS at their institute in Pakistan [15]. Hypertension and diabetes are the leading risk factor in patients having ACS with 70.4% and 51.2% respectively. Similarly, we had a huge bulk of patients admitted in our tertiary care hospital with ACS. The major risk factors in our study population was again hypertension (67.5%) and diabetes (46.2%).

A multi-centre study report a range of standardized in-hospital mortality rate of around 1.1–3.3% [16]. The standard bench mark set was 3.5%. In our study population, we had a mortality rate of 3.5% in 2019 and it jumped to 5.2% in 2020. The possible explanation could be that as the pandemic was ongoing, there were delayed presentation of patient to a hospital facility which might lead to worse outcomes. Bleeding is a major concern with coronary procedures. An annual incidence of bleeding with percutaneous intervention has increased exponentially from 1.8% to 5.8% from 2004 to 2014 [17]. Mehta et al. also reported bleeding rate in 2.4% of their patients [18]. A 90^th^ percentile bleeding rate set by ACC/AHA is around 1.4%. In our study, we noticed a bleeding rate of 1.15% in 2019 and 1.60% in 2020.

The appropriateness criteria for percutaneous intervention was around 99% in a report published in 2013 [19]. In our study, we achieved 80% appropriate PCI while 20% were possible appropriate in both the years making it almost 100%. When we observe appropriateness of PCI in a stable coronary disease, we found around 35% appropriateness in 2019 which fall down to 10% in 2020. The possible reason for this decline was the COVID-19 pandemic in which patients and physicians were afraid of having unnecessary exposure to the health care facilities.

Patients undergoing PCI had higher mortality in our study. This may be due to the heterogeneity of our group of patients with unstable angina, NSTEMI, and STEMIs. Secondly, all percutaneous interventions were recorded at the time of admission and not at the time of the onset of symptoms. A lower hemoglobin level and a higher creatinine level before PCI were also associated with mortality.

Length of hospital stay is a better efficacy predictor in NCDR registry. Surprisingly, the median length of hospital stay was 3 days in US patients [20]. Another study by Swaminathan et al. shows that outcomes of patients discharge in 2 days were similar to the patients who were discharge in 4 to 5 days [21]. We also had a shorter stay of our patients with percutaneous intervention. The median stay of our study patients were 2 days in 2019 and 1 day in 2020.

There were certain limitations in our study. Firstly, it is a single centre study using NCDR as a standard benchmark and the results cannot be generalized to the whole population. Secondly, there were some missing variables which were difficult to extract due to retrospective design. Thirdly, we cannot comment on the factors that caused delay in the PCI outside hospital.

This study helped us in identifying the difference of our population from other parts of the world and served as an important tool in recognizing and improving quality of care provided to the patients. Future studies are recommended on South Asian population. It can help in better understanding of the disease and health care centers can use it as a standard protocol in implementing quality of care provided to the patients with cardiovascular diseases.

## Conclusion

This study described the common and unique characteristics of patients with myocardial infarction representing population from a center in LMIC of South Asian region. Overall, the procedural performance measure and outcome metrics are up to the international benchmarks. Cultural, financial, and pandemic effects identified certain challenges. Consistency of the “performance” should be assessed continuously at the institute level for the better patient outcomes.

## Supporting information

S1 Table Comparison of characteristics of patients who survive versus non-survive. (DOCX)

S1 Data (XLSX)
