# Supplementary material for: Acute myocardial infarction from a lower-middle income country—A comprehensive report on performance measures and quality metrics using National Cardiovascular Data Registry
Source: PLoS One. 2023 Nov 15;18(11):e0294396. doi: 10.1371/journal.pone.0294396 (PMC10651007; doi:10.1371/journal.pone.0294396)
Supplement: S1 Table — (DOCX) [file pone.0294396.s001.docx]

**Supplementary table: Comparison of characteristics of patients who survive versus non-survive**

|  | ***Survivor***  ***N= 1477 (%)*** | ***Non-survivor***  ***N= 65 (%)*** | ***p-value^^*** |
| --- | --- | --- | --- |
| **Mean age ± S.D. (years)*^** | 61.5 ± 12.6 | 65.2 ± 12.8 | 0.02 |
|  |  |  |  |
| **Gender** |  |  |  |
| Male | 989 (66.9) | 53 (81.5) | 0.16 |
| Female | 482 (32.6) | 12 (18.4) |  |
|  |  |  |  |
| **Co-morbid** |  |  |  |
| Hypertension | 1001 (67.7) | 40 (61.5) | 0.29 |
| Diabetes | 682 (46.1) | 30 (46.1) | 0.99 |
| Prior history of cardiovascular disease* | 21 (1.4) | 3 (4.6) | 0.04 |
| Dyslipidemia | 341 (94.7) | 19 (5.3) | 0.25 |
|  |  |  |  |
| **Family history of ischemic heart disease** | 31 (2) | 0 (0) | 0.23 |
|  |  |  |  |
| **Smoking** | 121 (8.1) | 2 (3) | 0.13 |
|  |  |  |  |
| **Diagnostic corangiography** | 1469 (99.4) | 65 (100) | 0.55 |
| **Percutaneous intervention*** | 420 (28.4) | 43 (66.6) | 0.009 |
|  |  |  |  |
| **Systolic blood pressure (pre-procedure) – mmHg^** | 136 ± 14 | 142 ± 7.5 | 0.15 |
|  |  |  |  |
| **Laboratory parameters^** |  |  |  |
| Troponin (pre-procedure) – ng/ml | 9.4 ± 23.6 | 27.0 ± 34.0 | 0.10 |
| Hemoglobin (pre-procedure) – g/dL* | 12.9 ± 1.9 | 10.2 ± 1.9 | <0.001 |
| Creatinine (pre-procedure) – mg/dL* | 1.1 ± 0.6 | 1.8 ± 0.6 | 0.002 |
|  |  |  |  |
| **Cath lab indication** |  |  |  |
| ACS ≤24 hour | 1041 (70.4) | 7 (10.7) | 0.52 |
| ACS >24 hour | 168 (11.3) | 0 (0) | 0.29 |
| New onset angina (less than 2 months) | 103 (6.9) | 0 (0) | 0.42 |
| Worsening angina | 118 (7.9) | 1 (1.5) | 0.70 |
| Stable known CAD | 4 (0.2) | 0 (0) | 0.87 |
| Suspected CAD | 45 (3.0) | 1 (1.5) | 0.15 |
| Vulvular disease | 10 (0.6) | 0 (0) | 0.80 |
| Cardiac arrhythmia | 1 (100) | 0 (0) | 0.93 |
| Cardiomyopathy | 8 (100) | 0(0) | 0.82 |
| LV dysfunction | 10 (100) | 0(0) | 0.79 |
|  |  |  |  |

* P-value significant <0.05

^ independent sample t-test applied with level of significance <0.05

^^ chi-square test applied with level of significance of <0.05
